# I do not want to set my own price! Indirect effects of emotions and moderation effects of skepticism explain reduced use intentions towards participative pricing models

**DOI:** 10.1371/journal.pone.0275499

**Published:** 2023-02-14

**Authors:** Regina Wittstock-Lang, Magdalena Bekk, Matthias Spörrle

**Affiliations:** 1 Department of Business and Economic Psychology, Seeburg Castle University, Seekirchen am Wallersee, Austria; 2 Department of Marketing and Brand Management, University of Cologne, Köln, Germany; Universitat Jaume I, SPAIN

## Abstract

Participative pricing models (i.e., auction, reverse auction, pay-what-you-want) have grown in importance compared to classical, non-participative pricing models (i.e., fixed price, discount). This study examined (1) relative use intentions regarding different (non-) participative pricing models, (2) the emotional responses triggered by the pricing models and influencing consumers’ use intentions, and (3) the moderating role of individual skepticism in this context. A between-subjects experiment (*N* = 505) with five groups, manipulating participative (auction, reverse auction, and pay-what-you-want) and non-participative (fixed price and discount) pricing models, detected reduced use intentions towards participative compared to non-participative pricing models. Even though participative pricing models induced higher levels of positive as well as negative emotions, the effects via positive emotions (promoting use intentions) were weaker than the effects via negative emotions (mitigating use intentions). Skepticism towards participative pricing models enhanced negative emotions and decreased positive emotions. Practical applications should rely on enhancing positive emotions while simultaneously reducing negative emotions, as they exert independent effects. Skepticism of potential users should be mitigated in the early stages of the customer relationship, e.g., via trustworthiness triggers.

## Introduction

Imagine you are looking for a PC game to play and you come across the game “theHunter: Call of the Wild”. The atmospheric open world of the game is exactly what you were looking for. To purchase the game you have three options. The Humble Bundle platform (www.humblebundle.com) offers this game via pay-what-you-want. Simultaneously, you can buy the same game at a fixed price (www.mmoga.net) or a discount (www.instant-gaming.com). Which of these options are you going to choose? Although hypothetical in this case, consumers oftentimes have to choose between different pricing models. Thus, understanding the factors influencing consumer reactions towards different pricing models is important for managers and sellers in deciding which of these pricing models they will offer.

Digitalization and the increased use of participative (or sometimes also called interactive) pricing models on the internet are opening up new opportunities for designing purchase settings. Innovative pricing models such as reverse auctions or pay-what-you-want (PWYW) are increasingly being offered for services or products online. The success of portals such as eBay shows that buyers and sellers of new and used items are conducting auction-based business profitably on the internet. Customers on platforms such as MyHammer use reverse auctions to purchase service offers. Providers such as Humble Bundle use the internet to successfully offer pay-what-you-want pricing models for software. The internet allows buyers and sellers to quickly exchange, compare and shape price information. Even though participative pricing models are conceivable in physical market settings as well, the virtual setting of the digital economy is particularly suitable for them because a very large number of users can be addressed simultaneously and the number of online purchases is increasing. Thus, it seems reasonable to predict the growing applications of these pricing models along with the generally increasing digitalization of consumer settings. This research aims to elucidate potential affective and attitudinal determinants of customers having to choose between these pricing models.

There are two different use case scenarios in which the choice between different pricing models can emerge for the customer in real-world consumer settings. First, sometimes customers can choose between different pricing models for the same product within the same retailer. For example, on eBay, a mobile phone is offered at a fixed price (to buy immediately) or (possibly at a lower price) via an auction. Another example is software trainings on Neowin Deals (www.deals.neowin.net), which are offered on the same purchase channel for a fixed price ($17.93) or via pay-what-you-want. Thus, within the same channel or retailer, a customer can choose between different pricing options for the same product. Here, the use case of the customer having to choose between different pricing models for the same offer emerges on the side of the provider.

A second way a use case can emerge is on the customer’s side when they are provided with different pricing models via different channels for the same product. An example of this would be the above-mentioned game “theHunter: Call of the Wild”. The game is offered via pay-what-you-want (www.humblebundle.com), or at a fixed price (www.mmoga.net) or a discount (www.instant-gaming.com). Another example: The Yummy Organics Company (www.yummy-organics.de) offers shoppers the option to purchase a product via pay-what-you-want. The same product can be purchased from Amazon (www.amazon.de) at a fixed price. In examples like these, the use case emerges on the consumer’s side, who can choose to obtain the same product via different pricing models offered by different providers.

What variables are we examining to predict customer choices between pricing models? Existing research regarding participative and classical pricing models mainly consists of the analysis of the pricing models with reference to purchase-specific emotions [1, 2], motivation-related payment factors [3], and a behavioral analysis of consumers [4] irrespective of their individual attitudes. Thus, the overall affective tone in general and customer-specific attitudes towards these pricing models have not been addressed so far. This is unfortunate given the strong behavioral relevance of emotions and the practical benefits of customer group segmentation based on target-specific attitudinal variables. Moreover, a comparison regarding relative emotions and attitudes towards different pricing models is missing from the academic discourse. This makes it impossible to coherently compare affective states and their behavioral relevance between different pricing models.

In line with this, the breadth of models examined within one research paradigm is rather limited. Most prior research on participative pricing models was based on the analysis of one [3, 5–8] up to (in five cases) a maximum of three [9–13] pricing models. We argue that our current understanding of pricing model preferences would benefit from directly comparing more pricing models especially given the fact that (1) companies are more or less free to choose from a multitude of pricing models and (2) consumers then have to choose between different pricing models offered on the market. In terms of the methodological approach, some prior studies compared pricing models [14–17], but most do not apply randomized assignment. Only a total number of four papers [9–11, 13] compares a maximum of three pricing models and employs an experimental approach thus allowing for causal conclusions.

As a third aspect, in terms of personality-based characteristics, studies on pricing models lag behind other fields of business research [18]. Only recently, the field has started to examine general consumer traits such as the need for cognition [9] and altruism [16]. We want to add to this emerging promising trend by examining positive and negative emotions and a cognitive attitudinal variable specific to the stimulus examined, i.e., skepticism towards participative pricing models.

Our research is the first empirical evidence comparing consumers’ intentions to use different participative and non-participative pricing models. We contribute to prior research by showing (1) that participative pricing models, compared to classical, non-participative pricing models, want to be used differently by users, (2) that differences in consumers’ intentions to use a pricing model can be explained by different negative and positive emotions triggered by the pricing models, and (3) that consumers’ levels of skepticism influence whether consumers intend to use non-participative or a participative pricing models. We are the first to show that the intention to use a particular pricing model is explained and driven by the positive and negative emotions consumers feel towards the respective pricing model. These positive and negative effects are influenced by an individual trait, namely skepticism towards participative pricing models. With this research, we aim at increasing our understanding of consumers’ use intentions of participative and non-participative pricing models in terms of emotions and attitudes generally associated with these pricing models (i.e., not related to a specific purchase). Our findings are valuable to managers, in so far as they will help managers to understand that participative pricing models trigger both negative and positive emotions. Thus, managers wanting to apply participative pricing models, need to decrease negative emotions towards such pricing models and increase positive emotions (e.g., by creating a pleasant environment or excellent customer service). Furthermore, our findings help managers to understand individual attitudes, like skepticism, which influence intentions to use participative pricing models. Since such traits can be estimated automatically on an individual level, based, for instance, on social media likes [19] such variables could be used for automatic customer segmentation assigning specific pricing models to different user groups. Thus, managers can address consumers’ with high levels of skepticism online with non-participative pricing models, whereas consumers’ with low levels of skepticism are addressed with participative pricing models, in particular PWYW.

## Theoretical background and hypotheses

There are already numerous publications on the topic of pricing models, which describe and analyze the (dis)advantages of a single pricing model such as pay-what-you-want (PWYW), reverse auctions (REVA), and auctions (AUCT) [3, 7, 20, 21]. Auctions are market institutions with an explicit set of rules determining resource allocation and prices based on bids from the market participants. If a product has no standard value or is a rare, a price can be set by a bidder during an auction. PWYW and reverse auctions, where the buyer is involved in the entire pricing process, are also called participative pricing models [7, 22]. PWYW is the most researched participative pricing model. It allows the user to determine the price to be paid (including paying a price of zero) and there is no option for the seller to refuse to provide the service or product afterward. Hence, the focus is on the buyer as the person who makes the final price decision [7]. Contrasting participative pricing models, are the non-participative pricing models, such as fixed prices. A fixed price is a firmly defined price that may neither be exceeded nor undercut. The customer is aware of the price to be paid [23].

In pricing models in general, the focus is on the willingness to pay a certain price. However, with participative pricing models, the challenge for customers is that they have to decide on the price by themselves. This decision is not easy for every customer to make. Some researchers [24] explored, among other things, the issue of the price the customer is willing to pay and emphasized that customers cannot be expected to pay the maximum price in PWYW settings. Real preferences exist, but there is no incentive compatibility, i.e., it is not in the participants’ interest to reveal their own preference. A low offer in a PWYW system may signal not only that they are not interested at all in that product, but also that they are egotistic. If participants receive the cue that it is appropriate to offer a lot (a high anchor), they are willing to comply because they want to avoid sending a signal that they are selfish. However, other factors (such as emotions) can also be identified that may have an impact on consumer perception.

Only few studies so far, used experimental settings to directly compare non-participative and participative pricing models. Most of these few papers, compared only two (e.g., one participative, one non-participative) pricing models. Comparing PWYW to fixed prices [15, 25, 26], or PWYW to free sampling and discounts [27] or even comparing PWYW to another participative pricing model [10, 28], prior research could show that despite being popular participative pricing models can be challenging for users. The acceptance of pricing mechanisms is influenced by experience and knowledge and participatory pricing is more accepted by those who already have experience of it [10]. In our research, we provide for the first time the comparison of five pricing models (three of them participative pricing models) in a direct comparison.

Table 1 lists an overview of all existing studies that have experimentally compared at least one classical, non-participative with at least one participative pricing model. Our research continues this existing research. By comparing the existing studies in the table below, it is shown that there is no research comparable to ours. We illustrate that intention to use is influenced by emotions and personal attitudes (i.e., skepticism). To the best of our knowledge, we are the first to compare a wide variety of five pricing models and consumers’ use intentions of these pricing models together with consumers’ emotions and attitudes in an experimental setting.

**Table 1:** Literature overview.

Author (et al.) year	Number of pricing models	Types of pricing models	Study	Outcome (main contribution)	Affective states	Cognitive variables
Barone (et al.) 2017	2	pay-what-you-want, fixed price	Study 1	low-power consumers value pricing control afforded by pay-what-you-want to a greater extent than do high-power individuals.	no	yes
	2	pay-what-you-want (+ reference price), fixed price	Study 2	findings suggest that low-power participants valued the control in pay-what-you-want pricing models for its power regulation capabilities	yes	yes
Chandran and Morwitz 2005	2	Participative and fixed prive environments	Study 1	Price participation leads to higher intent to buy than economically equivalent fixed price conditions	no	yes
	2	Participative and fixed prive environments	Study 2	Price participation with perception of control increases purchase intent vs. fixed price environments	no	yes
	2	Bidding format “set your own price”	Study 5	Price participation with perception of control increases purchase intent vs. fixed price environments	no	yes
	2	Participative and fixed prive environments	Study 6	Perceived effort and task involvement don’t mediate the effect of price condition on purchase intent	no	yes
Di Domenico (et al.) 2022 [29]	2	Pick-your-price and fixed pricing model	Study 1	price transparency seems to lead to 1) more positive motives attributions to brands, 2) higher brand attitudes and 3) more willingness to pay a higher price for the product.	yes	no
	2	Pick-your-price and fixed pricing model	Study 2	setting a high default price has a detrimental effect on brand attributions, which, in turn, leads to more negative brand attitudes in the context of a PYP strategy.	yes	no
	2	Pick-your-price and fixed pricing model	Study 3	introducing price transparency in a PYP setting is also conducive to more revenues	no	no
	2	Pick-your-price and fixed pricing model	Study 4	consumers develop more positive brand attitudes and, in turn, higher willingness to purchase when the price transparency points to external CSR practices	yes	no
Gerpott (et al.) 2016	2	posted price, pay-what-you-want	Study 1	price-related buyer attitudes related to customer choice of pay-what-you-want offers. Practitioners should carefully reflect on consumer attitudes evoked by the pay-what-you-want mechanism in case of considering its introduction and in designing its details.	no	yes
Haws and Bearden 2006 [30]	2	price-discovery vs. price-posted mechanisms	Study 1	Higher prices paid relative to other consumers will trigger stronger negative fairness judgments than seller, time, and/or price-setter differences. With price-setter differences, higher prices paid relative to others trigger weaker negative fairness judgments than seller, time, and/or consumer differences.	no	yes
	2	price-discovery vs. price-posted mechanisms	Study 2	t consumers are more willing to accept prices that they themselves played a role in setting	no	yes
	2	price-discovery vs. price-posted mechanisms	Study 3	proximal prices have more impact when consumers feel they are at a disadvantage	no	yes
Hinz (et al.) 2011	2	Fixed threshold price setting vs. adaptive threshold price setting	Study 1	The adaptive threshold price policy is more profitable than the fixed threshold price policy	yes	no
	2	Fixed threshold price setting vs. adaptive threshold price setting	Study 2	The participative nature of dynamic pricing mechanisms mitigates the negative perceptions of price discrimination.	yes	no
Kim (et al.) 2009	2	Pay-what-you-want (PWYW) vs. regular fixed price	Study 1	Prices Paid in PWYW are significantly higher than zero	no	yes
	2	Pay-what-you-want (PWYW) vs. regular fixed price	Study 2	Prices Paid in PWYW are significantly higher than zero	no	yes
	2	Pay-what-you-want (PWYW) vs. regular fixed price	Study 3	Prices Paid in PWYW are significantly higher than zero, also exceed regular (fixed) prices	no	yes
Kim (et al.) 2014	2	sampling, pay-what-you-want	Study 1	differences in perceived promotional characteristics and relevant performance measures, such as trial and repeat purchases by new customers for pay-what-you-want	no	no
	2	pay-what-you-want, discount	Study 2	pay-what-you-want appears to be an intermediate solution between sampling and discounts that may yield the highest revenues	no	no
Krämer (et al.) 2017	2	pay-what-you-want, name-your-own-price	Study 1	pay-what-you-want and name-your-own-price can be successfully used to endogenously price discriminate	no	no
Rathore (et al.) 2022	3	pay-what-you-want, pick-your-price, fixed price	Study 1	higher purchase intention for pay-what-you-want and pick-you-price than for fixed price	no	yes
	3	pay-what-you-want, pick-your-price, fixed price	Study 2	pricing effort mediates the relationship between pay-what-you-want and purchase intentions for high need for cognition consumers and pick-your-price and purchase intention for low need for cognition consumers	no	yes
	3	pay-what-you-want, pick-your-price, fixed price	Study 3	prices paid by high need for cognition individuals would be similar to the market price	no	yes
	3	pay-what-you-want, pick-your-price, fixed price	Study 4	increased profitability of participative pricing strategies even when the price paid per unit is not significantly different from the fixed price.	no	yes
Schmidt (et al.) 2015	1	pay-what-you-want	Study 1	PWYW can be viable on a monopolistic market, but it is less successful as a competitive strategy because it does not drive traditional posted-price sellers out of the market.	no	no
Schröder (et al.) 2015	2	pay-what-you-want, mark-off-your-own-price, fixed price, rebate	Study 1	prices are significantly lower and more customers choose a price of zero in the mark-off-your-own-price compared to the pay-what-you-want condition	no	no
Sulser (2021)	3	pay-per-minute, pay-what-you-want, fixed price	Study 1	acceptance of pricing mechanisms is influenced by experiential learning	no	no
Viglia (et al.) 2019	1	pay-what-you-want	Study 1	positive relationship between the regular price of the meal and pay-what-you-want amounts	no	no
	1	pay-what-you-want	Study 2	consumers pay more before consumption only when sources of uncertainty can be resolved. Implementing pay-what-you-want after service consumption mostly resolves this uncertainty.	no	no
Wagner (et al.) 2022	3	pay-what-you-want, name-your-own-price, fixed price	Study 1	perceived control and the element of uncertainty are crucial in determining how participative pricing mechanisms affect satisfaction with pricing and payment morale.	yes	no
	2	pay-what-you-want, name-your-own-price	Study 2	Pay-what-you-want produces higher satisfaction with pricing and lower pain when paying. External reference prices appear to increase consumers’ willingness to pay	yes	no
Wang (et al.) 2021	3	pay-what-you-want, pick-your-price, fixed price	Study 1	pick-your-price increases purchases and profits by enhancing pricing control (relative to fixed price) but alleviates pricing effort (relative to pay-what-you-want).	no	no
	3	pay-what-you-want, pick-your-price, fixed price	Study 2	relative to a fixed price, pay-what-you-want undermines, whereas pick-your-price increases, purchase intentions, regardless of price level. Pick-your-price elicits greater pricing control but does not affect pricing effort, relative to a fixed price.	no	yes
	3	pay-what-you-want, pick-your-price, fixed price	Study 3	participants indicated higher purchase intentions in pick-you-price (vs. fixed-price) condition and lower purchase intentions in pay-what-you-want (vs. fixed price) condition	no	yes
	2	fixed price, pick-your-price	Study 4	when pricing effort increases, consumers are less likely to purchase, even if the pricing strategy offers more pricing control. When pricing control decreases, consumers also show weaker purchase intentions, even when the pricing strategy evokes the same level of pricing effort	no	yes
	2	fixed price, pick-your-price	Study 5	pricing control and effort significantly mediated the effect of pricing strategies on purchase choices.	no	yes
Wang (et al.) 2022	2	pay-what-you-want, fixed price	Study 1	Customers have a favorable attitude toward participative pricing and its opposite effect on purchase intentions. Customers experience fatigue, they are even more likely to avoid effortful decisions like determining the price for a product offered with pay-what-you-want.	yes	no
	2	pay-what-you-want, fixed price	Study 2	Observed negative effects of pay-what-you-want (vs. fixed price) on purchase intentions, especially among fatigued individuals.	yes	no
	2	pay-what-you-want, fixed price	Study 3	Pay-what-you-want resulted in significantly fewer sales, with a more substantial negative effect later in the day, as customers’ fatigue levels increased.	yes	no
	2	pay-what-you-want, fixed price	Study 4	Fatigued customers perceive pay-what-you-want pricing as even more effortful, and this demand ultimately reduces their purchase likelihood.	yes	no
THIS STUDY	5	Auction, reverse auction, pay-what-you-want, fixed price, discount	Study 1	Consumers‘ show lower use intentions for participative (vs. non-participative) pricing models. The effect of pricing models on use intentions is explained by negative and positive emotions independently. Strong skepticism increases negative emotions, and decreases positive emotions and use intentions.	yes	yes

Emotional responses may be important in participative pricing models. Current research mostly refers to and examines one emotion in one pricing model [2, 31–33], to show that the paid price may be related to, for example, discomfort or overload. Emotions are an integral part of bidders’ decisions and strategies [2]. Emotional effects influence the bidder [31] (for similar findings regarding emotional arousal in auctions) and play a role in the relationships among various constructs surrounding price. It was demonstrated that incidental arousal increases bidding and final prices, without individuals’ awareness. Enjoyment was found to play a role, along with cognitive variables (such as, e.g., price-quality associations), in respondents’ reactions to price [32]. Based on the findings that decisions in auctions are influenced by emotions, we assume that emotions also play a role in other participative pricing models.

Emotions, like overload, shame and arousal have an impact on consumer behavior. Positive and negative emotions, for example, are related to perceived price fairness and consumer switching behavior [33]. Perceived fairness is significantly related to emotions, and while emotions similarly influence behavioral responses, they also mediate the relationship between perceived price fairness and behavioral responses. These findings suggest that emotions have a mediating impact on consumer behavior [33]. Our research assumes a similar process. To the best of our knowledge, no research comparatively examines multiple participative and non-participate pricing models on emotions and addresses both positive and negative emotions acting simultaneously.

Whether a consumer uses a participative pricing model can not only be influenced by emotions, but also by social influences [34, 35], the individual personality of the person [36, 37] or cognitive mechanisms [38]. Thus, personality-based characteristics might help explain why different individuals respond differently towards the same pricing model. Prior research, examining a bidding platform comparable to eBay, could show that personality has a meaningful predictive power in explaining bidding behavior, but only for female participants [39]. In the case of PWYW, traits such as social desirability and social approval are related to the pricing decision in PWYW [16]. Social desirability contributes to making a good impression and self-appreciation. Social forces such as a concern for the individual’s public image influence pricing decisions in the PWYW context [16].

### Participative pricing models

There are several participative pricing models. Within this study, we focus on reversed auctions (REVA), auctions (AUCT), and pay-what-you-want (PWYW). In reverse auctions, the roles of price setter and creator of the auction mechanism are reversed compared to those in the classic auction: sellers are the bidders, and the goal is to push the price down. In this case, we have a single buyer and several suppliers [23]. Reverse auctions are well established in the business-to-business environment and are mainly used by business customers to find suppliers. They focus on price and not on quality or service [40].

With the pricing model PWYW, buyers independently decide what price they want to pay and whether they want to pay. No price is prescribed to the buyer. In field experiments, it was shown that people are willing to pay to maintain their positive self-image when using PWYW. The results show that, frequently, fewer consumers choose to purchase a product through PWYW than when the price is fixed and low. The buyer feels bad when the price falls below the reasonable price and is more likely to abandon the purchase altogether than to underpay via PWYW [14].

There are many providers using the PWYW pricing model: restaurants such as “Weinerei” in Berlin, “Wiener Deewan” in Vienna or “Terrabite” in Seattle offer PWYW for their lunch buffet. It can also be found in theatres such as “Pure reFORM” in Poland, museums like the “Lehmbruck Museum” in Germany, zoos such as the “Alwetterzoo Münster” in Germany, software developers like “Adblock” [7] or massage courses such as “Massage Around the World” [41]. A live stream of Jules Evans and Michael Pollans discussing the latter’s book “This is your mind on plants” is available as a PWYW model on the Royal Institution’s website (https://www.rigb.org/whats-on/events-2021/august/public-this-is-your-mind-on-plants). Tickets can be purchased by interested parties for £0–£21.14.

### Hypotheses

To develop our hypotheses (1–2), we assume that participative pricing models are generally used less frequently than the traditional fixed price or discount models, thus resulting in generally lower use intentions. This assumption is based on prior research, which could show that customers are more likely to choose a lower fixed price than to underpay via pay-what-you-want [14]. We assume that this holds for different non-participative pricing models (i.e., fixed price and discount). We further assume that this finding can be applied to other participative pricing models, because customers dislike being in the situation of being seen as freeloaders or misjudging the "right" price.

**Hypothesis 1.** The use intention of a fixed price is higher than the use intention of (a) an auction, (b) a reverse auction, and (c) pay-what-you-want.
**Hypothesis 2.** The use intention of a discount is higher than the use intention of (a) an auction, (b) a reverse auction, and (c) pay-what-you-want.

There is already some evidence that emotions can play a role, for example, in auctions. In classical auctions, reverse auctions and online versions of these, emotions, such as anticipated frustration, occur and have consequences for subsequent bidding processes [2]. Excitement during an auction can seem positive for the seller (because a higher price is likely to be achieved) and negative for the bidder (because he may overbid and pay more). Thus, the impact depends from which point of view the auction is viewed [42].

In PWYW, consumers may pay more than they intend, viewed from the background that they pay a price they deem to be suitable to save face [15] as they may be under the scrutiny of other consumers. They do not want to be seen as freeloaders in front of other customers and will try to maintain their reputation. Such social aspects within PWYW situations could trigger negative emotions towards PWYW.

As consumers are at risk of overpaying when using participative pricing models [15, 42] it seems reasonable to assume that negative feelings arise when being confronted with participative pricing models. Therefore, we hypothesize that participative pricing models have higher negative emotionality (compared to non-participative pricing models, such as fixed price or discounts).

**Hypothesis 3.** Negative emotions are stronger with (a) an auction, (b) a reverse auction, and (c) pay-what-you-want than with fixed prices.
**Hypothesis 4.** Negative emotions are stronger with (a) an auction, (b) a reverse auction, and (c) pay-what-you-want than with discounts.

Prior research could show that some consumer groups particularly like to use participative pricing models [25] as consumers use intention is in some cases motivated by bargain hunting and "value shopping" [3]. Thus, we propose that participative pricing models have higher positive emotionality compared to the non-participative fixed price models. We assume this is the case, as customers will experience more positive feelings due to the cheaper purchase they can make when using participative pricing models.

**Hypothesis 5.** Positive emotions are stronger with (a) an auction, (b) a reverse auction, and (c) pay-what-you-want than with fixed prices.

Regarding discounts, prior research could show that, compared to fixed prices, discounts have a positive and significant effect on positive emotions [43]. This can be explained due to the potential price saving, which is usually perceived positively. As consumers do not want to be seen as freeloaders in front of other customers and will try to maintain their reputation, they will pay a price suitable to save face when using participative pricing models [15]. Thus, consumers tend to overpay when using participative pricing models [15, 42] and are thus likely to pay more than the discount price would be. Thus, we assume that discounts elicit more positive emotions, due to a lower price being paid, compared to participative pricing models.

**Hypothesis 6.** Positive emotions are stronger with discounts than with (a) an auction, (b) a reverse auction, and (c) pay-what-you-want.

Focusing on individual trait variation, we focused on skepticism towards participative pricing models. An existing research [44] distinguished between trait and state skepticism. This means that consumers vary in their predisposition to being skeptical and that skepticism can be generated by situational variables that trigger a (temporary) state of skepticism. Generally speaking, a consumer could have a skeptical personality (attitude: as in our research). Regardless of any information, a skeptical person will tend to question things. A person may become more skeptical when they encounter new pricing models or interactive products and is thus confronted with specific unfamiliar stimuli. We assume that skepticism towards participative pricing models is an object-specific attitude. To find out how skeptical our participants are, we used and modified existing scales [45–47] to reliably capture the level of skepticism towards participative pricing models.

**Fig 1:**
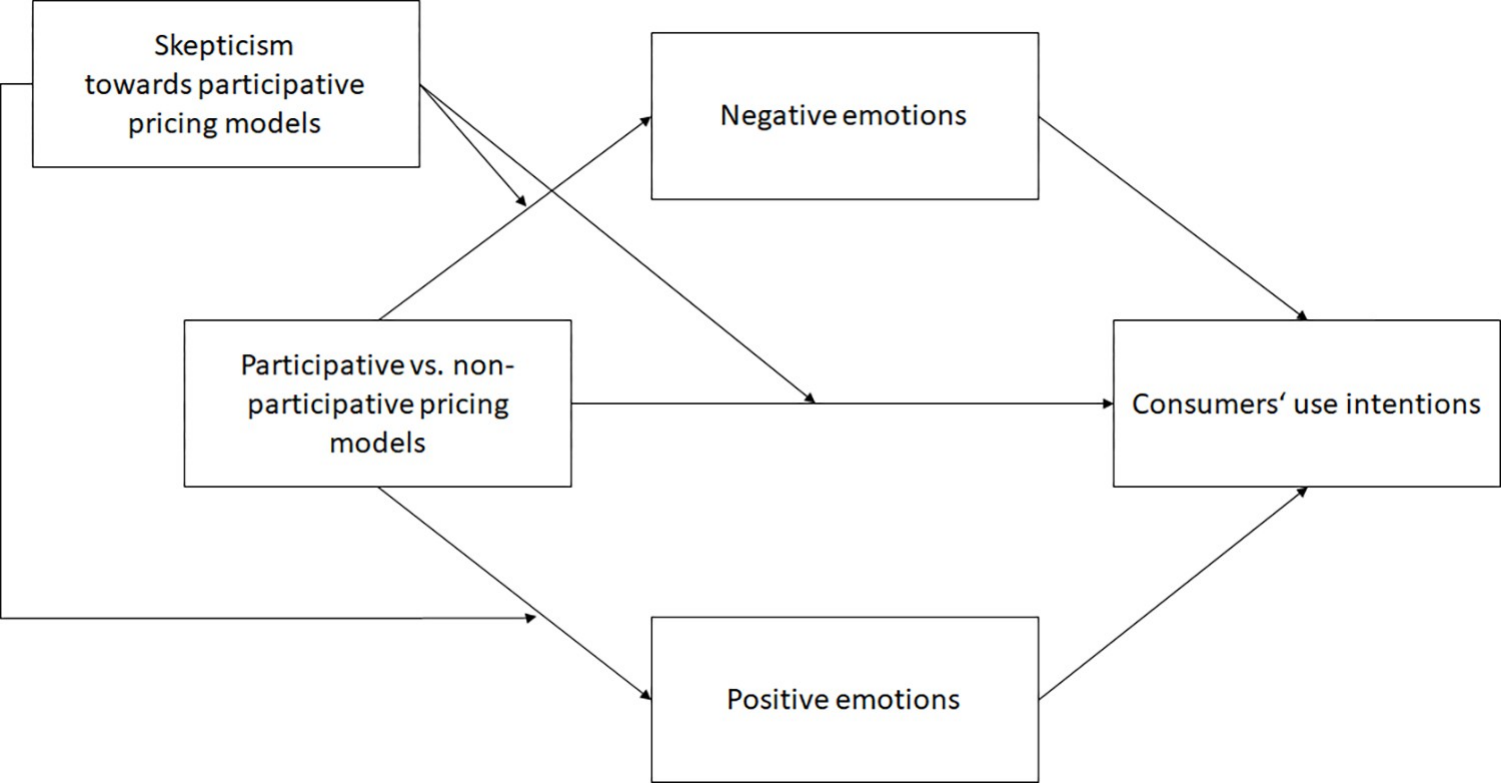
Conceptual framework.

It has already been shown in other domains [48] that skepticism can mitigate positive effects on consumer responses. We used this reasoning to further assume enhancing effects of skepticism on negative effects on consumer responses. A skeptical attitude towards participative pricing models should aggravate the consumer’s us intentions and negative emotional response. We did not differentiate between pricing models because we assumed that the amplification or attenuation applies equally to all participative pricing models. Therefore, we developed Hypotheses 7 to 9 and assumed that skepticism would increase the effects on the emergence of negative emotions and weaken the effects on use intentions and positive emotions. See Fig 1 for a conceptual framework of our hypotheses.

**H7.** Skepticism towards participative pricing models reduces consumers’ use intentions of participative pricing models (compared to non-participative pricing models).
**H8.** Skepticism towards participative pricing models increases consumers’ negative emotions towards participative pricing models (compared to non-participative pricing models).
**H9.** Skepticism towards participative pricing models reduces consumers’ positive emotions towards participative pricing models (compared to non-participative pricing models).

## Materials & methods

### Participants and procedure

Using the software Unipark (https://www.unipark.com/), an online experiment was created for data collection. The survey was sent, via e-mail, to individuals aged 18 and over in Germany. The link to the experiment was distributed via e-mail within the authors’ own network as well as through the authors’ university network. The link was also posted online on various platforms such as XING (https://www.xing.com), Facebook (https://www.facebook.com), and Clickworker (https://www.clickworker.com). This study was approved by the Ethics Committee of Seeburg Castle University. Participants aged 18 and above could take part in the online experiment between September and October 2018. The processing time for completing the online experiment was 25 minutes on average. In addition to assessing positive and negative emotions, intentions to use, and skepticism about participative pricing models, the post-experiment survey section contained several additional sections that are not relevant to this study. Demographic data was collected at the end. The final data was exported to IBM SPSS for analysis.

Overall, 530 responses were collected, although the data from 25 participants was excluded prior to analysis because of missing data. The final sample size was *N* = 505 (*n*_auction_ = 105, *n*_reverse auction_ = 99, *n*_PWYW_ = 102, *n*_discount_ = 102, *n*_fixed price_ = 97). The final sample consisted of 50.9% female and 49.1% male participants, with an average age of 38.5 years (*SD* = 12.08).

### Experimental design and stimulus material

For our study, we used a randomized, experimental, one-factorial design with five different pricing models as between-subjects conditions: Auction (AUCT), reverse auction (REVA), pay-what-you-want (PWYW), discount (DISC), and fixed price (FIPR). Each participant was randomly assigned to one of these five pricing models. After reading the instructions for the experiment, participants read a description of the pricing model. For example, for the p*ay-what-you-want* pricing model: “You pay whatever price you think is appropriate for a product or service. No price is set. You decide completely independently what the product or service is worth to you. This is usually a time-limited pricing model.” For the full descriptions of all pricing models, see S1 File.

### Measures

As mediators, we assessed negative and positive emotions toward the respective pricing model. We measured negative emotions using three items on 6-point Likert scales ranging from “not true at all” (numerical label “0”) to “completely true” (numerical label “5”) (α = 0.82 and ω = 0.82), sample item: “[pricing model] scares you”. We measured positive emotions towards the respective pricing model using three items on 6-point Likert scales ranging from “not true at all” (numerical label “0”) to “completely true” (numerical label “5”) (α = 0.83 and ω = 0.83), sample item: “[pricing model] makes you happy.” For a list of all items used in this study, see S1 Table. Prior to further analyses, we computed the mean value for these and all following construct measures, which were then z-transformed.

As moderator, we assessed individual skepticism towards participative pricing models. We used five items on 6-point Likert scales ranging from “not true at all” (numerical label “0”) to “completely true” (numerical label “5”) (α = 0.80 and ω = 0.79), sample item: “You are generally critical of any new pricing model.”.

For measuring our dependent variable, i.e., intention to use the pricing model, we used three items on 6-point Likert scales ranging from “not at all”/ “not true at all” (numerical label “0”) to “extremely”/“completely true” (numerical label “5”) (α = 0.82 and ω = 0.82), sample item: “Would you like to use [pricing model] again/for the first time?”.

## Results

Descriptive statistics as well as Pearson and Spearman correlations for the mediating, dependent, and moderating variables can be found in Table 2. The high convergence of Pearson and Spearman correlations as well as kurtosis and skewness levels close to zero indicate the normality of our variables.

**Table 2:** Descriptive table and correlations for variables assessed.

	*M*	*SD*	*Skewness*	*Kurtosis*	(1)	(2)	(3)	(4)
(1) Negative Emotion	2.01	0.93	0.79	-0.06	(0.82)	-.40**	-.50**	.37**
(2) Positive Emotion	2.94	0.93	-0.08	-0.35	-.38**	(0.83)	.64**	-.21**
(3) Use Intention	3.47	1.03	-0.38	-0.53	-.51**	.63**	(0.82)	-.19**
(4) Skepticism	3.00	0.83	-0.18	0.07	.33**	-.21**	-.18**	(0.80)

Note: *M* = Mean, *SD* = Standard deviation, above main diagonal Pearson’s r, below main diagonal Spearman’s rho, Cronbach’s Alpha in main diagonal.

### Comparison of participative versus non-participative pricing models with regard to consumers’ use intentions and emotions

First, to test our hypotheses 1 to 6, we compared each of the three participative pricing models (i.e., auction, reverse auction, pay-what-you-want) with each of the two classical, non-participative pricing models (i.e., fixed price, discount) on use intention of the pricing model, as well as negative and positive emotions toward the pricing model (see Table 3 for means and standard deviations).

**Table 3:** Mean values for the five pricing models on emotions and use intention.

	FIPR	AUCT	REVA	PWYW	DISC
	*M* (*SD*)	*M* (*SD*)	*M* (*SD*)	*M* (*SD*)	*M* (*SD*)
Negative Emotion	1.82 (0.85)	2.13 (0.92)	2.08 (0.95)	2.28 (0.98)	1.73 (0.83)
Positive Emotion	2.64 (0.74)	2.77 (0.87)	2.78 (0.83)	2.98 (0.91)	3.53 (0.99)
Use Intention	3.71 (0.95)	3.03 (1.02)	3.07 (0.91)	3.43 (1.04)	4.13 (0.84)

Note.

*M* = mean values, *SD* = standard deviations in brackets; FIPR = Fixed Price. AUCT = Auction. REVA = Reverse auction. PWYW = Pay-what-you-want. DISC = Discount.

Looking at consumers’ use intentions first, our results show that there are significant differences between the five pricing models, *F*(4, 500) = 23.67, *p* < .001, η^2^ = .16. We used pairwise planned contrasts to compare each of the participative pricing models with each of the non-participative pricing models. Our results show that in line with H1a, H1b, and H1c, the use intention of a fixed price model (*M* = 3.71, *SD* = 0.95) is higher than the use intention of an auction (*M* = 3.03, *SD* = 1.02, *p* < .001, Cohen’s *d* = .69), higher than the use intention of a reverse auction (*M* = 3.07, *SD* = 0.91, *p* < .001, *d* = .69) and also higher than the use intention of a pay-what-you-want (PWYW) pricing model (*M* = 3.43, *SD* = 1.04, *p* = .04, *d* = .28). In line with H2a, H2b, and H2c, the use intention of a discount (*M* = 4.13, *SD* = 0.84) is higher than the use intention of an auction (*p* < .001, *d* = 1.18), a reverse auction (*p* < .001, *d* = 1.21), and PWYW (*p* < .001, *d* = .75). Thus, our hypotheses H1a-c and H2a-c are confirmed. Consumers’ show higher use intentions for non-participative compared to participative pricing models.

Looking at consumers’ negative emotions toward the pricing models, our results show that there are significant differences between the five pricing models, *F*(4, 500) = 6.12, *p* < .001, η^2^ = .05. We again used pairwise planned contrasts to compare each of the participative pricing models with each of the non-participative pricing models. In line with H3a, H3b, and H3c, our results show that compared to a fixed price model (*M* = 1.82, *SD* = 0.85) an auction (*M* = 2.13, *SD* = 0.92, *p* = .02, *d* = .35), a reverse auction (*M* = 2.08, *SD* = 0.95, *p* = .05, *d* = .29), and a PWYW pricing model (*M* = 2.27, *SD* = 0.98, *p* < .001, *d* = .49) induce significant stronger negative emotions. In line with H4a, H4b, and H4c, an auction (*p* < .01, *d* = .45), a reverse auction (*p* = .01, *d* = .39), and PWYW (*p* < .001, *d* = .59) induce stronger negative emotions than a discount (*M* = 1.74, *SD* = 0.83). Thus, our hypotheses H3a-c and H4a-c are confirmed. Consumers’ show higher negative emotions for participative compared to non-participative pricing models.

Looking at consumers’ positive emotions toward the pricing models, our results show that there are significant differences between the five pricing models, *F*(4, 500) = 16.34, *p* < .001, η^2^ = .12. We again used pairwise planned contrasts to compare each of the participative pricing models with each of the non-participative pricing models. Our results show that positive emotions are not significantly lower for a fixed price model (*M* = 2.64, *SD* = 0.74) compared to an auction (*M* = 2.77, *SD* = 0.87, *p* = .23, *d* = .17), or a reverse auction (*M* = 2.79, *SD* = 0.83, *p* = .19, *d* = .19). Thus, H5a and H5b are rejected. In line with H5c, a PWYW pricing model (*M* = 2.98, *SD* = 0.91) induces stronger positive emotions than a fixed price, *p* = .01, *d* = .42. In line with H6a, H6b, and H6c, a discount (*M* = 3.53, *SD* = 0.99) induces stronger positive emotions than an auction (*p* < .001, *d* = .81), a reverse auction (*p* < .001, *d* = .81), or PWYW (*p* < .001, *d* = .57). Thus, our hypotheses H6a-c are confirmed. Consumers’ show stronger positive emotions for discounts compared to participative pricing models. But a PWYW pricing model induces stronger positive emotions compared to a fixed price (H5c).

### Mediation via negative and positive emotions

We used model 4 within the PROCESS Syntax [49] in IBM SPSS to test for parallel mediation through negative and positive emotions. We assume that the effect of participative versus non-participative pricing models on consumers’ use intentions is explained through negative and positive emotions towards these pricing models. We ran two parallel mediation models: Both were identical in that they used use intentions as the criterion and negative and positive emotions as mediators. Regarding the pricing models, the first mediation model compared fixed prices against the three participative pricing models, using fixed prices as the baseline. The second mediation model compared discounts against the three participative pricing models, using discounts as the baseline.

#### Fixed price baseline model

Comparing fixed prices against the three participative pricing models, our results show that negative (β = -.29, *p* < .001) and positive (β = .52, *p* < .001) emotions significantly influenced consumers’ use intentions. Negative emotions explained the effect of participative versus fixed price pricing models on consumers’ use intention for auctions versus fixed pricing (indirect effect *ab* = -.10, 95% confidence interval [95%CI] = [-.18; -.02]), and for PWYW versus fixed pricing (*ab* = -.14, 95%CI = [-.24; -.06]), but not for reverse auctions versus fixed pricing (*ab* = -.08, 95%CI = [-.17; .00]). Positive emotions explained the effect of participative versus fixed price pricing models on consumers’ use intention for PWYW versus fixed pricing (*ab* = .19, 95%CI = [.07; .33]). Neither for auctions (*ab* = .08, 95%CI = [-.05; .20]) nor for reverse auctions (*ab* = .08, 95%CI = [-.04; .21]) versus fixed pricing did positive emotions explain the effect of participative versus fixed price pricing models on consumers’ use intention.

#### Discount baseline model

Comparing a discount pricing model against the three participative pricing models, our results show that negative (β = -.16, p < .001) and positive (β = .60, p < .001) emotions significantly influenced consumers’ use intentions. Negative emotions explained the effect of participative versus discount pricing models on consumers’ use intention for all three participative pricing models: Auctions versus fixed pricing (*ab* = -.07, 95%CI = [-.13; -.02]), reverse auctions versus discount (*ab* = -.06, 95%CI = [-.12; -.02]), and PWYW versus discount (*ab* = -.09, 95%CI = [-.16; -.04]). Positive emotions explained the effect of participative versus discount pricing models on consumers’ use intention for all three participative pricing models: Auctions versus discount (*ab* = -.49, 95%CI = [-.67; -.32]), reverse auctions versus discount (*ab* = -.48, 95%CI = [-.66; -.30]), and PWYW versus discount (*ab* = -.35, 95%CI = [-.55; -.18]).

#### Robustness check

As a robustness check, we run our analyses also including biological sex and chronological age as covariates. We detected one main effect of these covariates in the model (i.e., a negative effect of chronological age on the emergence of positive emotions). Most importantly, our findings regarding the effects of participative versus non-participative pricing models on consumers’ use intentions and the mediating effects via negative and positive emotions did not change in terms of direction or (non)significance. Providing evidence of the robustness of our findings, these analyses indicate that our findings do not vary when age or sex are statistically excluded from the model. Thus, our analyses provide a relatively high level of robustness.

### Moderated mediation: Testing skepticism towards participative pricing models as moderator

We used model 58 within the PROCESS Syntax [49] in IBM SPSS to test for skepticism towards participative pricing models as the moderator within the above tested parallel mediation models through negative and positive emotions towards these pricing models. We ran two moderated parallel mediation models: Both were identical in that they used consumers’ use intentions of the pricing model as the criterion and negative and positive emotions towards the pricing model as mediators. Regarding the pricing models, the first moderated mediation model compared fixed prices against the three participative pricing models, using fixed prices as the baseline. The second moderated mediation model compared discounts against the three participative pricing models, using discounts as the baseline. In both models, we included skepticism as moderator on the paths between pricing models and emotions as well as between pricing models and use intentions.

#### Fixed price baseline model

Comparing fixed prices against the three participative pricing models, our results show—in line with the prior analyses—that negative (β = -.25, *p* < .001) and positive (β = .50, *p* < .001) emotions significantly influenced consumers’ use intentions. There was no significant effect of skepticism on consumers’ use intentions (β = .14, *p* = .14), nor on negative emotions (β = -.04, *p* = .76) or positive emotions (β = .10, *p* = .38).

**Fig 2:**
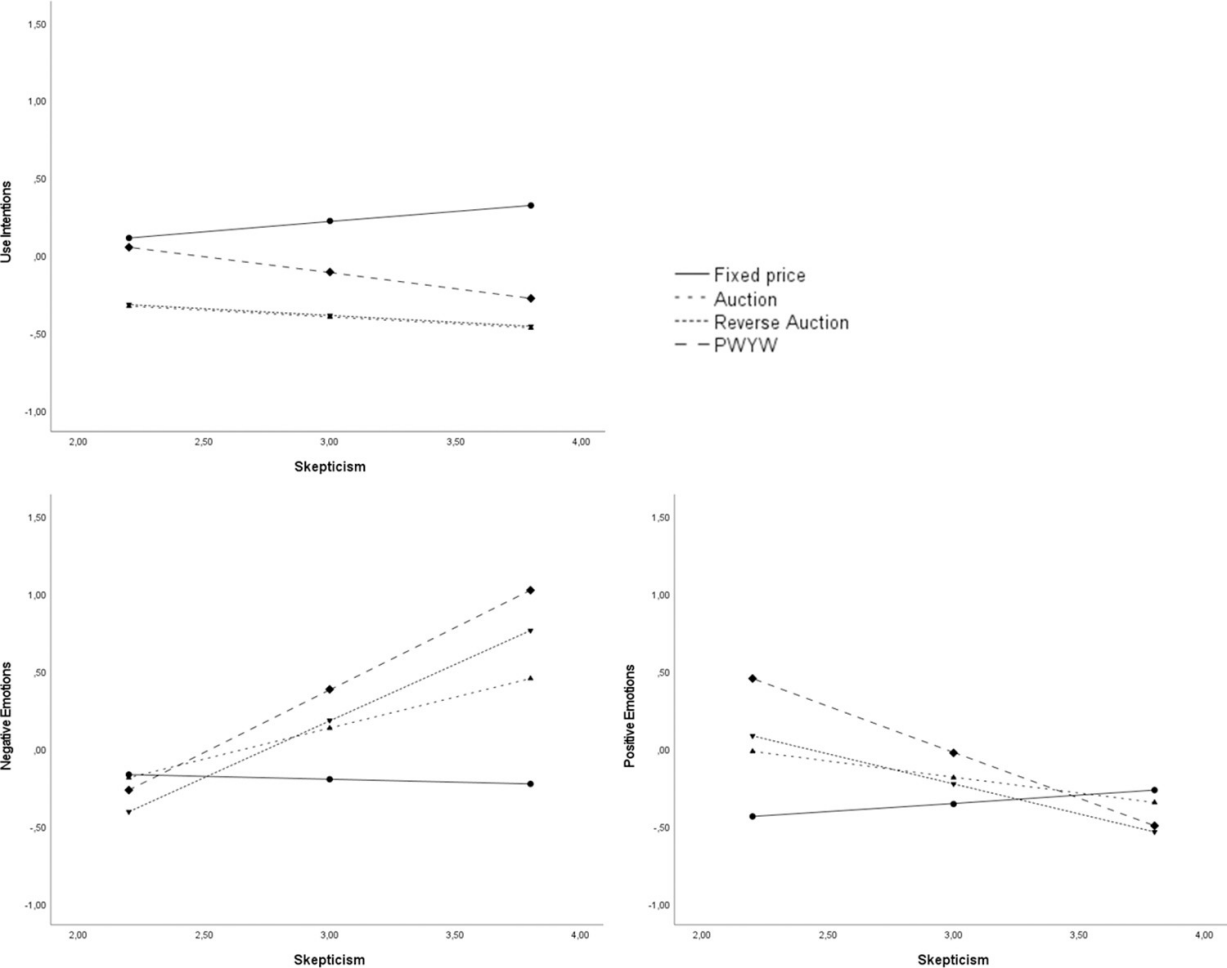
Simple slopes for the fixed price model with skepticism towards participative pricing models as moderator.

There was, however, a significant interaction between skepticism and PWYW (vs. fixed price) on use intention (β = -.34, *p* = .01). Consumers’ use intentions did not differ for PWYW compared to fixed price, if they experienced low levels of skepticism (β = -.06, *p* = .71). However, if consumers’ experienced medium or high levels of skepticism a significant negative effect occurred between PWYW (vs. fixed price) and use intentions (skepticism_Mean_ β = -.33, *p* < .001; skepticism_Mean+1SD_ β = -.60, *p* < .001). See Fig 2 for the simple slopes analyses of the interactions.

There were also significant interactions between skepticism and all three participative (auction: β = .43, *p* = .01; reverse auction: β = .76, *p* < .001; PWYW: β = .84, *p* < .001, all vs. fixed price) pricing models on negative emotions. Consumers’ felt the same level of negative emotions for the three participative pricing models then for the fixed price if they experienced low levels of skepticism. With increasing levels of skepticism towards participative pricing models, consumers’ also felt significantly more negative emotions when faced with a participative (vs. fixed price) pricing model (for skepticism_Mean+1SD_: auction: β = .67, *p* < .001; reverse auction: β = .98 *p* < .001; PWYW: β = 1.25, *p* < .001).

There were also significant interactions between skepticism and all three participative (auction: β = -.31, *p* = .01; reverse auction: β = -.49, *p* < .001; PWYW: β = -.70, *p* < .001, all vs. fixed price) pricing models on positive emotions. Consumers’ felt the same level of positive emotions for the three participative pricing models then for the fixed price model if they experienced high levels of skepticism. For low levels of skepticism, consumers’ felt significantly more positive emotions when faced with the participative (vs. fixed price) pricing model (for skepticism_Mean-1SD_: auction: β = .42, *p* = .03; reverse auction: β = .52 *p* < .001; PWYW: β = .89, *p* < .001).

#### Discount baseline model

Comparing discounts, as our second non-participative pricing model, against the three participative pricing models, our results show—in line with the prior analyses—that negative (β = -.11, *p* = .01) and positive (β = .58, *p* < .001) emotions significantly influenced consumers’ use intentions. There was no significant effect of skepticism on consumers’ use intentions (β = .07, *p* = .35), nor on positive emotions (β = -.08, *p* = .47). There was a significant positive effect of skepticism on negative emotions (β = .44, *p* < .001).

**Fig 3:**
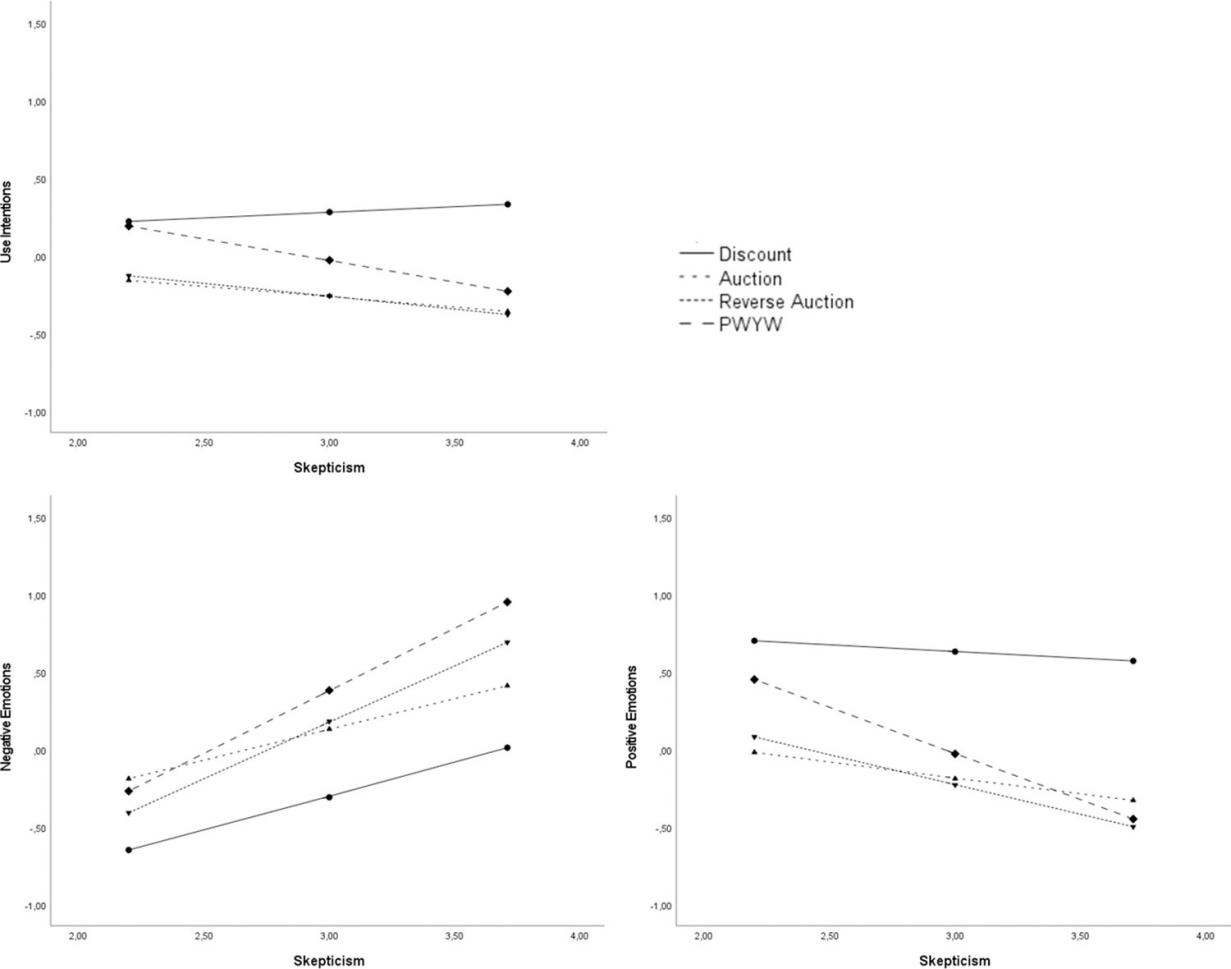
Simple slopes for the discount model with skepticism towards participative pricing models as moderator.

There were, however, significant interactions between skepticism and all three participative (auction: β = -.21, *p* = .05; reverse auction: β = -.24, *p* = .03; PWYW: β = -.35, *p* < .001, all vs. discount) pricing models on consumers’ use intentions. Consumers’ use intentions of the participative pricing models compared to discounts decreased with increasing levels of skepticism (for skepticism_Mean-1SD_: auction: β = -.38, *p* < .001; reverse auction: β = -.35, *p* < .001; for skepticism_Mean+1SD_: auction: β = -.69, *p* < .001; reverse auction: β = -.71, *p* < .001; PWYW: β = -.56, *p* < .001). There was no significant difference in consumers’ use intentions between PWYW and discounts for low levels of skepticism (β = -.03, *p* = .82). See Fig 3 for the simple slopes analyses of the interactions.

There were also significant interactions between skepticism and reverse auctions (β = .29, *p* = .05) as well as PWYW (β = .37, *p* = .01) pricing models compared to discounts on consumers’ negative emotions. With increasing levels of skepticism, consumers’ felt significantly more negative emotions towards reverse auctions (for skepticism_Mean+1SD_: β = .69, *p* < .001) and PWYW (for skepticism_Mean+1SD_: β = .95, *p* < .001) compared to discounts.

There was also a significant interaction between skepticism and PWYW (vs. discount) on positive emotions (β = -.51, *p* < .001). Consumers’ levels of positive emotions did not differ for PWYW compared to discounts, if they experienced low levels of skepticism (β = -.25, *p* = .17). However, if consumers’ experienced medium or high levels of skepticism, consumers’ felt significantly lower levels of positive emotions for PWYW compared to discounts (skepticism_Mean_: β = -.66, *p* < .001; skepticism_Mean+1SD_: β = -1.03, *p* < .001).

## Discussion

This paper contributes to the pricing literature by demonstrating differing use intentions regarding participative and non-participative pricing models, respectively, and by elucidating the emotional antecedents of these intentions as a function of individual skepticism.

Compared to fixed prices, lower use intentions towards participative pricing models were demonstrated. Use intentions were independently determined by negative as well as positive emotions, with the latter having a substantially stronger effect. Even though participative pricing models tended to evoke higher levels of positive emotions (in most cases not reaching significance) they, without exception, also elicited higher levels of negative emotions (in most cases reaching significance) with the latter effects being systematically stronger than the former ones. Use intentions of participative pricing models were influenced by negative and positive emotions at the same time, thus indicating that both affective tendencies must be considered simultaneously when addressing emotional antecedents of use intentions for pricing models.

Regarding individual skepticism towards participative pricing models, our research confirmed that with increasing levels of skepticism negative emotions towards participative (vs non-participative) pricing models increased, and use intentions as well as positive emotions towards participative (vs non-participative) pricing models decreased. This was particularly the case for PWYW pricing models. We found a consistent pattern of results not anticipated and not derivable by the scientific status quo: The enhancing effect of skepticism on the emergence of negative emotions is stronger than its mitigating effect on the emergence of positive emotions. Thus, skepticism shapes both approach-oriented positive emotions as well as avoidance-oriented negative emotions, but the effects on motivational avoidance processes seem to be consistently stronger [50]. Thus, skepticism emerged as a double hazard as it decreased the effects on use intentions and positive and increased the effects on negative emotions.

### Implications for theory and practice

This paper introduces the simultaneous consideration of both positive as well as negative emotions in the analyses of pricing model use intentions. Previous research in other domains, such as technology [51], food innovation acceptance [52], or entrepreneurial intention [53], has been able to implement this approach successfully and has demonstrated predictive simultaneous validity of positively and negatively valenced affective constructs in terms of behavioral intentions. Despite these promising findings from other domains, this approach has been neglected in pricing model research. In terms of pricing model research, some studies have examined, for instance, negative emotions [54] but did not consider positive emotions. Our research provides the first evidence that both affect directions should be considered simultaneously (i.e., the presence of positive emotions is not identical with low levels of negative emotions in terms of predictive value) in future pricing model research because it demonstrates independent predictive validity of positive as well as negative affect.

Previous research describing the use and implementation of participative pricing models [28, 55] showed the differences between participative pricing models [20], and consumer responses to these models [5, 56–58]. So far only a few studies compared only one single participative pricing model (such as PWYW) with fixed prices [7]. Thus, the comparison between several traditional, non-participative pricing models and several participative pricing models reveals a research gap so far. This broad comparative approach with several pricing models allows for further generalizations and demonstrates three important contributions to the existing research on participative pricing models.

First, the study showed that the use intention of the participative pricing models (compared to non-participative pricing models) is generally decreasing. We assume that reverse auctions are more likely to be found in the business-to-business process [55] and therefore do not appeal to the ordinary consumer. In comparison, PWYW is more important in B2C markets and prior research could show that this participative participatory pricing model is used in many different areas [59]. The fact that PWYW lowers use intentions is important to managers thinking about applying PWYW in their businesses. Based on these results, we can assume that consumers may not want to decide on their own price or that pricing by consumer participation may not be desired. Participative pricing models might create uncertainty for consumers.

Second, we focused on negative and positive emotions and showed that the intention to use participative pricing models is influenced by the evoked negative and positive emotions. In the case of PWYW pricing, we found that consumers are not unanimous but rather ambivalent about whether they perceive this pricing model as positive or negative. Both negative and positive emotions influence PWYW use intention, but the effect of negative emotions is stronger. Existing studies have already shown that discomfort can arise with PWYW and that consumers can go so far as abandoning the purchase process [15]. Thus, our research follows and extends existing research and shows that PWYW is an emotional pricing model, with consumers exhibiting split emotions.

Third, we added skepticism towards participative pricing models to our model. With increasing skeptical attitudes towards participative pricing models, the effect on negative emotion is strengthened and the effect on positive emotion is weakened. These results show a new approach to that of previous research and suggest that consumers’ attitudes play consistent and independent roles in emotions towards participative pricing models. Since traits such as the Big Five can be estimated automatically on an individual level, based, for instance, on social media likes [19] such variables could be used for automatic customer segmentation assigning specific pricing models to different user groups. Thus, managers can address consumers’ with high levels of skepticism online with non-participative pricing models, whereas consumers’ with low levels of skepticism are addressed with participative pricing models, in particular PWYW.

Our research fills many gaps in the existing literature. We compared several participative pricing models with non-participative pricing models to prove that the use intention of participative pricing models is lower. However, this use intention can be (or is, in fact) influenced by consumers’ emotions. The fact that personal attitudes, like skepticism, also affect use intention is a novel finding in this context and indicates how many aspects need to be taken into account in connection with the use of participative pricing models.

This paper raises the bar for comparing pricing models. Most research on pricing models examines consumer reactions, for instance, customer experience, within a single pricing model [3, 6–8]. Until now, only four papers have followed an extended comparative approach and have compared a maximum of three pricing models [10, 11, 27]. Our research extends this fruitful comparative approach by comparing five pricing models and indicates systematic differences between these models within the same methodological approach. Thus we can demonstrate the need for multiple pricing model comparisons in future research. Especially in terms of emotional responses, we found that different participative pricing models result in distinctive responses compared to non-participative pricing models, illustrating the need to compare several participative pricing models within the same research paradigm.

Our research extends the rather limited examination of trait-based attitudinal characteristics of potential users. Existing research has indeed started to address personal characteristics (e.g., social [16]; altruism [60]; egoism [61]). However, this research, which has exclusively focused on willingness to pay as a criterion and has only examined processes regarding a single pricing model, has only examined the main effects of these person-based characteristics, but not potential moderations by these variables. Our research thus extends previous approaches to examining person-based characteristics within pricing model research by elucidating the need to consider the potential moderation (or experimentally: interaction) effects of these person-based variables. Our significant findings demonstrate the predictive relevance of considering these effects for future research and theory building.

Through our research, we were able to show that customer emotions have an impact on the use intention regarding participative pricing models, which can be an important insight for providers. An already existing attitude strengthens or weakens the emotions of the consumer. Consumers may not be clear about what they expect from participative pricing models and what benefits they might have. Here, it is important to inform customers and to strengthen and accompany customers when using participative pricing models.

Companies should recognize the mindset of their customers and understand how to influence their customers’ emotions, as this creates the opportunity to use participative pricing models in a meaningful way. When offering PWY, providers need to recognize and influence customers’ emotions, as we could register both negative and positive emotions with this pricing model. In this case, possibilities for doing so would be to gain the trust of the customer, to create transparency in the use of participative pricing models, or to create an environment in which the user feels comfortable (such as by providing good service or friendly staff).

Auctions and reverse auctions, on the other hand, should generally be offered with more caution. Companies should be aware that auctions tend to reinforce negative emotions. Reverse auctions show negative reinforcement, especially among skeptics, which can be important information in negotiations in the business-to-business environment. If the promotion of the pricing model takes these points into account and also appeals to its customers on an emotional level, it could be a way to attract customers to participative pricing models. So far, we have investigated skepticism towards participative pricing models and have already formulated the explanation that these attitudes can influence emotions and the use intention of these models. We show that providers need to mitigate skepticism about participative pricing models by, for example, creating a pleasant and familiar environment or by using trustworthy endorsers and testimonials.

In summary, we can say that participative pricing models are already finding numerous different applications on the one hand, but that there is still further potential for participative pricing models on the other. Providers must consider that emotions and personal attitudes have independent and interactive effects on consumers’ use intention.

### Limitations and future research

Even though our study provides a helpful paradigm to compare affective and conative responses between different pricing models, some of its limitations also provide fruitful opportunities for future research. Examining auction, reverse auction, PWYW, discount and fixed price pricing models, we addressed the essential classical and participative pricing models. We did not consider other participative pricing models with smaller areas of application such as name-your-own-price (which is identical to PWYW, except for the fact that the seller must agree to the price before the transaction can take place) or pay-per-minute pricing. Our paradigm is compatible with these (so far) niche models, but did not consider them.

Moreover, other attitudes (besides skepticism towards participative pricing models) or personality traits might serve as additional promising moderators. Personality traits such as risk aversion and emotional stability (from the Big Five), or belief in a just world [62] could be incremental fruitful consumer-based boundary conditions. Possible approaches to further research could be person-side determinants in conjunction with skepticism. Publications on the acceptance of innovations [63] can be found in the literature. One possible question would be how the acceptance of innovative pricing models and skepticism are related, and whether distinct effects on use behavior can be demonstrated. Looking at determinants of skepticism, another approach would be to examine how skepticism varies according to individual characteristics and histories [64] intercultural aspects, knowledge, and values.

Our analyses provide valuable insights by pointing to systematic effects of positive and negative affect; future research could build on these promising findings by examining distinct (negative and positive) emotions such as fear and anger or enjoyment and pride. Moreover, since we only used self-reported emotions we could only assess the conscious emotional component (i.e., the feeling). Future research should also address the unconscious component of emotions towards pricing which also shapes decision processes [65]. We also used consumers’ self-reported intentions to use a particular pricing model. Future research should further validate our results, by examining real-time consumer behavior, for example by using field experiments in which pricing models are varied between different points in time [7].

Our approach was general and did not consider specific product categories. Further research approaches could address which product categories might be particularly well suited for specific pricing models and distinguish between non-participative and participative pricing models to show providers which product category should rather be offered via an participative pricing model.

Another approach would be to extend our methodological approach to dynamic pricing models and show whether there are differences in consumer reactions towards dynamic compared to participative pricing models. Dynamic pricing is defined as an attempt by a seller to force a customer to pay the highest price they are willing to spend [66]. Even though dynamic and participative pricing models are substantially different (e.g., in terms of transparency towards the customer and influenceability by the customer), they have some shared features (e.g., a non-static price). Therefore, it would be interesting to examine how consumers respond to dynamic pricing compared to participative pricing models (e.g., in terms of emotions). The critical point here is that the process must be transparent, which is difficult to capture in dynamic pricing as companies adjust prices for products or services based on current market demand, and consumers are usually unaware that they are in a dynamic pricing process.

## Conclusion

With this research, we aim at increasing our understanding of consumers’ use intentions of participative and non-participative pricing models in terms of emotions and attitudes generally associated with these pricing models (i.e., not related to a specific purchase). Consumers’ intention to use a particular pricing model is explained and driven by the positive and negative emotions consumers feel towards the respective pricing model. These positive and negative effects are influenced by an individual trait, namely skepticism towards participative pricing models. Our results suggest that participative pricing, which offers consumers the opportunity for interaction, is sometimes not desired by consumers and evokes negative emotions, in particular among consumers who experience high levels of skepticism towards participative pricing models. While the associated flexibility generates positive emotional effects, participative pricing models also generate negative effects that tend to be stronger. The positive effects are unquestionably an opportunity that can be exploited for market launches, but managers need to find ways of reducing negative emotions and skepticism if the use of participative pricing models is planned.

## Supporting information

S1 File Descriptions of pricing models used in experiment. (DOCX)

S1 Table Variable definition (for each question, the price model queried in each case is used). (DOCX)
