# Supplementary material for: I do not want to set my own price! Indirect effects of emotions and moderation effects of skepticism explain reduced use intentions towards participative pricing models
Source: PLoS One. 2023 Feb 14;18(2):e0275499. doi: 10.1371/journal.pone.0275499 (PMC9928114; doi:10.1371/journal.pone.0275499)
Supplement: S1 File — (DOCX) [file pone.0275499.s001.docx]

**Supporting information**

**S1 Descriptions of pricing models used in experiment**

**Reverse auction**: An auction is held during a certain period of time. A person who wants something makes a corresponding request (usually online) to several potential providers of that good or service. These potential suppliers then each submit an offer for the provision of the good or service independently of each other (i.e., without knowledge of the offers of the others). The buyer selects the most suitable (usually the cheapest) of these offers. The Internet platform "MyHammer", for example, works according to this principle: You make an inquiry for a service and several people (mostly craftsmen there) can independently submit an offer to you for the fulfilment of the work. You have a certain amount of time to do this. After that, no more offers can be submitted.

**Auction**: An auction is held during a specified time period. There is a single product (or only a few) and several bidders (often more bidders than products/bids. The highest bid wins and thus gets the product.

**Pay-what-you-want**: You pay whatever price you think is appropriate for a product or service. No price is set. You decide completely independently what the product or service is worth to you. This is usually a time-limited pricing model.

**Fixed price**: Probably the most common pricing model. There is a clearly specified price for a service or product. The price cannot be negotiated. The fixed price always applies.

**Discount**: For a limited period of time, you have the opportunity to purchase a product for a reduced price. Here, the currently valid reduced price is always presented, and the other "base price" (= usual price) is usually presented as the reference price. This is usually a time-limited pricing. (The price reduction with higher purchased quantity, the quantity discount, is not meant herewith).
