# Supplementary material for: I do not want to set my own price! Indirect effects of emotions and moderation effects of skepticism explain reduced use intentions towards participative pricing models
Source: PLoS One. 2023 Feb 14;18(2):e0275499. doi: 10.1371/journal.pone.0275499 (PMC9928114; doi:10.1371/journal.pone.0275499)
Supplement: S1 Table — (DOCX) [file pone.0275499.s002.docx]

**Supporting information**

**S2 Table. Variable definition (for each question, the price model queried in each case is used).**

| **Research Variables** | **Original items in German** | **Items translated into English** | **Answer options** | **α** | **ω** |
| --- | --- | --- | --- | --- | --- |
| **Negative emotion** | 1. Bei [Preismodell] beschleicht Sie ein Gefühl des Unwohlseins.  2. [Preismodell] machen Ihnen Angst.  3. [Preismodell] geben Ihnen ein schlechtes Gefühl. | \| 1. When using [pricing model] you feel uneasy. \|  \|  \|  \| \| --- \| --- \| --- \| --- \| \| 2. [pricing model] scares you. \|  \|  \|  \| \| 3. [pricing model] gives you a bad feeling. \|  \|  \|  \| | **not true at all ( = 0 )**  **completely true ( = 5 )** | **0.82** | **0.82** |
| **Positive emotions** | 1. [Preismodell] machen Sie glücklich.  2. [Preismodell] machen Ihnen Spaß / bereiten Freude.  3. [Preismodell] geben Ihnen ein gutes Gefühl. | \| 1. [pricing model] makes you happy. \| \| --- \| \| 2. [pricing model] is fun. \| \| 3. [pricing model] gives you a good feeling. \| | **not true at all ( = 0 )**  **completely true ( = 5 )** | **0.83** | **0.83** |
| **Use intention pricing model** | 1. Wie gerne würden Sie [Preismodell] (wieder / erstmalig nutzen?  2. [Preismodell] würden / werden Sie wieder / erstmalig nutzen.  3. [Preismodell] würden / werden Sie regelmäßig nutzen. | \| 1. Would you like to use [pricing model]  (again/for the first time)? \| \| --- \| \| 2. Would you use/will use [pricing model] again  /use for the first time. \| \| 3. Would you use/  will you use [pricing model] regularly. \| | **not at all ( = 0 )**  **extremely ( = 5 )**  **not true at all ( = 0 )**  **completely true ( = 5 )** | **0.82** | **0.82** |
| **Scepticism towards interactive pricing models** | 1. Sie stehen generell neuen Preisfindungsmodellen kritisch gegenüber.  2. Sie stehen neuen Preisfindungsmodellen (wie Reverse Auction / Pay-what-you-want) skeptisch gegenüber.  3. Neue Preismodelle machen den Kaufprozess komplexer.  4. Über neue Preisfindungsmodelle einzukaufen ist so kompliziert, sodass Sie manchmal gar nicht verstehen wie der Prozess eigentlich funktioniert.  5. Sie setzen sich mit neuen Preisfindungsmodellen gar nicht auseinander, weil es zu lange dauert sich damit zu beschäftigen und herauszufinden, was der Nutzen daraus sein kann. | 1. You are generally critical of any new pricing models.  2. You are sceptical about new pricing models (like REVA/ PWYW)  3. New pricing models make buying processes more complex.  4. Shopping using new pricing models is so complicated that sometimes you don’t even understand how the process actually works.  5. You do not even deal with new pricing models because it takes too long to deal with it and to find out what the benefits can be. | **not true at all ( = 0 )**  **completely true ( = 5 )** | **0.80** | **0.79** |
